# Latest Advances in Obesity Treatment by Regulating Adipocyte Thermogenesis

**DOI:** 10.1155/ppar/1742256

**Published:** 2026-06-21

**Authors:** Tao Nie, Jianbin Chen, Zixin Wang, Liufeng Mao

**Affiliations:** ^1^ School of Basic Medicine, Hubei University of Arts and Science, Xiangyang, China, hbuas.edu.cn; ^2^ The First Affiliated Hospital, Guangdong Pharmaceutical University, Guangzhou, China, gdpu.edu.cn; ^3^ Neurological Research Institute of Integrated Traditional Chinese and Western Medicine, The First Affiliated Hospital, Guangdong Pharmaceutical University, Guangzhou, China, gdpu.edu.cn

**Keywords:** adipocyte thermogenesis, beige adipocyte, brown adipocyte, obesity, UCP1

## Abstract

Obesity is driven by chronic energy imbalance and is associated with multiple metabolic diseases. As adipocyte thermogenesis contributes to energy expenditure, it is being actively investigated as a possible target for obesity treatment. This review summarizes recent progress in the molecular regulation of adipocyte thermogenesis, focusing mainly on preclinical mechanistic evidence from cellular and animal studies, while discussing clinical and human data only where available. We review emerging regulators within the cAMP pathway, the PRDM16‐PPAR*γ* axis, and mitochondrial quality control and metabolism, together with other pathways that modulate brown and beige adipocyte function. We also discuss their metabolic effects in experimental obesity settings. Nevertheless, the therapeutic relevance of these findings remains uncertain because much of the evidence is still preclinical, and because translation is complicated by species differences, adipose depot heterogeneity, and the limited number of validated human studies. This review therefore offers an updated synthesis of recent mechanistic advances while emphasizing the key challenges that must be addressed before these insights can be reliably translated into obesity therapies.

## 1. Introduction

Obesity, a condition of pervasive global prevalence, is fundamentally characterized by a chronic imbalance between energy intake and expenditure. Obesity is associated with a spectrum of serious metabolic complications, including Type 2 diabetes, cardiovascular diseases, and metabolic dysfunction‐associated steatotic liver disease (MASLD) [1]. Although reducing caloric intake remains a cornerstone of obesity management, long‐term maintenance of weight loss is often difficult because of compensatory biological adaptations and the frequent recurrence of weight regain [2, 3]. This therapeutic limitation has intensified interest in strategies that increase energy expenditure. In this context, non‐shivering thermogenesis in adipose tissue has emerged as a physiologically relevant mechanism that not only contributes to temperature homeostasis but also influences body weight change [4–7].

Adipose tissue is a heterogeneous and highly plastic organ, including white adipose tissue (WAT), brown adipose tissue (BAT), and inducible beige adipocytes within WAT depots [8, 9]. White adipocytes, dedicated to energy storage and endocrine signaling, accumulate in both subcutaneous and visceral adipose depots. Critically, the expansion of visceral WAT is strongly associated with adverse metabolic outcomes, whereas subcutaneous WAT (sWAT) expansion has been paradoxically linked to improved insulin sensitivity in both humans and rodents [10–14]. In contrast to the prominent interscapular BAT depot in rodents and human infants, adult humans possess metabolically active thermogenic adipocytes in supraclavicular, cervical, paravertebral, perirenal, and other anatomical regions, with many depots showing molecular features consistent with a mixture of classical brown and beige adipocytes rather than a single uniform BAT type [15, 16].

**Figure 1:**
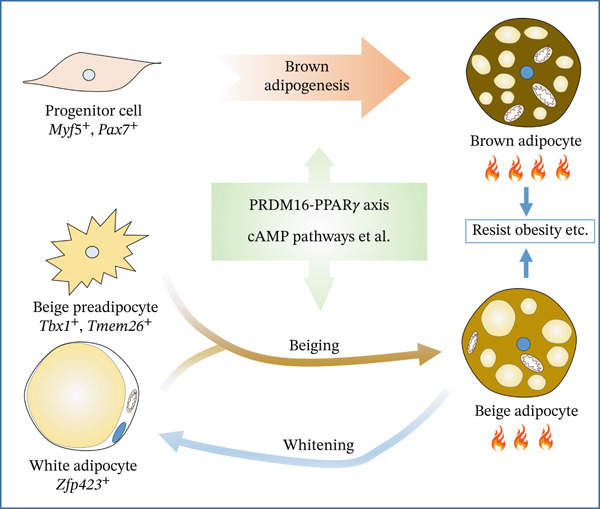
Thermogenic adipocytes include brown and beige adipocytes. Brown adipocytes are classical thermogenic fat cells derived mainly from *Myf5^+^* (myogenic factor 5‐positive) and *Pax7^+^* (paired box 7‐positive) progenitor cells. Beige adipocytes are inducible thermogenic adipocytes that can arise either from dedicated precursors within white adipose tissue or through reprogramming of white adipocytes in response to environmental or hormonal stimuli such as cold exposure. Beige adipocytes may also revert toward a white adipocyte‐like state under certain conditions. Both brown and beige adipocytes contribute to adaptive thermogenesis through high mitochondrial content and expression of UCP1 (uncoupling protein 1). The figure was created using PowerPoint.

The thermogenic function resides in brown and beige adipocytes. Brown adipocytes originate from *Myf5^+^* and *Pax7^+^* progenitors [17, 18], while beige adipocytes can emerge within WAT through a process termed “browning” or “beiging” from resident precursors or be reprogrammed from white adipocytes (Figure 1) [19]. The exceptional thermogenic capacity of brown and beige adipocytes is attributed to their unique cellular architecture, featuring a multitude of mitochondria and several small lipid droplets [20]. The central effector of this heat production is Uncoupling Protein 1 (UCP1), which is densely expressed on the inner mitochondrial membrane [21]. UCP1 dissipates the proton electrochemical gradient generated by the oxidative phosphorylation (OXPHOS) complex in the electron transport chain, thereby uncoupling substrate oxidation from ATP synthesis and releasing energy as heat [22, 23]. The integrity of mitochondrial function is therefore paramount for adipocyte thermogenesis. In obesity, adipose tissue often exhibits mitochondrial dysfunction, including aberrant fission and fusion dynamics, cristae remodeling, and oxidative stress, which collectively suppress UCP1 mediated activity and impair the tissue’s metabolic health [24, 25].

The regulation of adipocyte thermogenesis is mediated by an interconnected network of molecular pathways, including sympathetic and *β*‐adrenergic signaling, thyroid hormone action, mitochondrial substrate cycling, calcium handling, creatine‐dependent futile cycling, and transcriptional regulators such as PGC1*α* and p38 MAPK [26–28]. These pathways have been extensively investigated because they provide candidate entry points for therapeutic intervention.

Recent reviews have examined thermogenic adipose tissue from several important but distinct perspectives (Table 1). For example, prior work has reviewed the mechanisms and drivers of obesity‐associated whitening of brown and beige adipose tissue, emphasizing the contributions of diet, aging, inflammation, hypoxia, angiogenic impairment, and vascular rarefaction to thermogenic decline [29]. Other reviews have focused on the secretory and endocrine functions of BAT, particularly BAT‐derived metabolites and batokines as mediators of inter‐organ communication and systemic metabolic regulation [30]. In parallel, therapy‐oriented reviews have summarized pharmacological and cell‐based approaches for increasing the activity or abundance of brown and beige adipocytes, including *β*3‐adrenergic agonists, transplantation strategies, and stem cell‐based interventions [31]. More recently, attention has also turned to the regulation of adipokines and batokines, including nutraceutical and flavonoid‐related approaches [32], as well as to the concept that adipose tissue may retain an epigenetic memory of obesity after weight loss, thereby contributing to persistent dysfunction and rebound weight gain [33].

**Table 1:** Positioning of the present review relative to recent representative reviews in thermogenic adipose tissue and obesity.

Recent review	Main focus	Gap relative to the present review	Distinct contribution of the present review
An insight into brown/beige adipose tissue whitening (2023)	Pathophysiology of BAT and beige fat whitening in obesity.	Focused mainly on causes and features of whitening rather than a systematic update of newly identified direct regulators of thermogenesis.	Provides an updated synthesis of new molecular regulators and pathways that directly modulate adipocyte thermogenesis, with in vivo obesity relevance.
BAT‐derived metabolites and their role in regulating metabolism (2024)	Endocrine role of BAT and BAT‐derived metabolites and batokines.	Emphasized secreted metabolites and systemic crosstalk, with less attention to the broader intracellular regulatory network governing thermogenesis.	Integrates intracellular signaling, transcriptional, mitochondrial, and metabolic regulators with obesity‐associated thermogenic dysfunction.
Thermogenic fat as a new obesity management tool: From pharmaceutical reagents to cell therapies (2024)	Therapeutic exploitation of thermogenic fat.	Primarily intervention‐oriented; less focused on consolidating the latest mechanistic discoveries that underlie thermogenic regulation.	Offers a mechanism to translation framework linking new regulators to therapeutic opportunities and limitations.
Regulation of adipokines and batokines, including nutraceutical perspectives (2025)	Adipokines, batokines, and modulation by phytochemicals or flavonoids.	Concentrated mainly on secreted factors and nutraceutical regulation.	Broadens the discussion to include direct, nonsecretory thermogenic regulators, adipocyte identity, and obesity‐linked dysfunction.

Despite these important advances, an updated and dedicated synthesis of the newly identified molecular regulators that directly control adipocyte thermogenesis and show physiological relevance in obesity in vivo remains limited. Accordingly, the present review is intended to complement rather than duplicate earlier reviews by focusing specifically on recent mechanistic discoveries, primarily from 2023 to 2025, and by integrating them into a broader framework that connects molecular thermogenic regulation, adipose dysfunction in obesity, reversibility versus persistence of thermogenic impairment, and translational potential in human metabolic disease.

Thus, the novelty of the present review lies in providing an updated mechanistic synthesis of newly identified thermogenic regulators with demonstrated physiological relevance in obesity, while also highlighting their implications for adipose plasticity and translational intervention.

## 2. Methodological Approach

This narrative review was designed to summarize recent molecular advances in the regulation of adipocyte thermogenesis with relevance to obesity treatment. The guiding question of this review was the following: which newly identified molecular regulators of adipocyte thermogenesis may have potential relevance for obesity?

Relevant studies were identified through literature searches in PubMed, supplemented by screening the reference lists of relevant original articles and recent reviews. Search terms included the combinations of “adipocyte thermogenesis,” “brown adipocyte,” “beige adipocyte,” and “obesity.” We primarily considered studies published from 2023 to 2025, while including earlier landmark studies where necessary to provide conceptual background. This timeframe was chosen to update recent progress beyond our previous summary of the field covering studies published from 2021 to 2023 [34].

Study selection and prioritization were guided by three criteria: (1) identification of novel regulators or pathways, (2) direct effects on adipocyte thermogenic function, and (3) physiological relevance to obesity. Greater emphasis was placed on studies with in vivo evidence, whereas in vitro mechanistic studies were included to clarify molecular mechanisms and human studies were discussed where available and directly relevant. As a narrative review, this article is intended to provide a structured synthesis of recent mechanistic and translational findings.

## 3. New Regulators in Cyclic Adenosine Monophosphate (cAMP) Signaling Pathway (Transcriptional and Post‐Transcriptional Regulation, Preclinical and Human Evidence)

**Figure 2:**
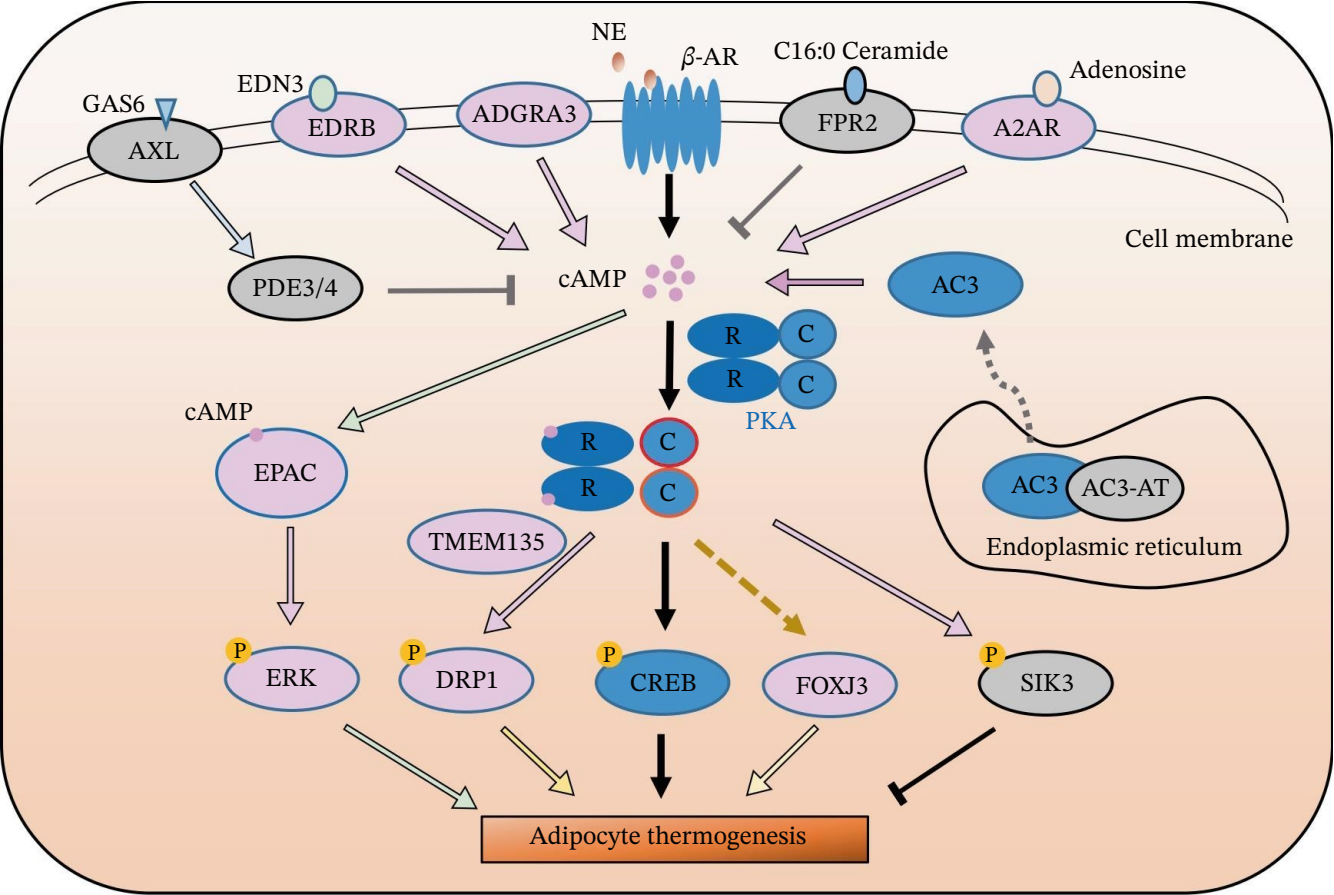
Genes and signaling modules involved in cyclic adenosine monophosphate (cAMP)‐dependent regulation of adipocyte thermogenesis. This schematic summarizes key stimulatory and inhibitory regulators of the cAMP‐PKA (protein kinase A) signaling pathway in thermogenic adipocytes. ADGRA3 (adhesion G protein‐coupled receptor A3) signaling activates the Gs‐cAMP‐PKA‐CREB (cAMP response element‐binding protein) axis. Extracellular adenosine or selective A2A receptor agonists increase intracellular cAMP levels, whereas C16:0‐ceramide‐activated FPR2 (formyl peptide receptor 2), the GAS6‐AXL‐PDE3/4 (growth arrest‐specific 6–AXL RTK–phosphodiesterase 3/4) module, and AC3‐AT, an endoplasmic reticulum‐tethered interactor of adenylyl cyclase 3, reduce cAMP and suppress PKA activity. In the EDN3 (endothelin 3)‐EDNRB (endothelin receptor type B) pathway, cAMP activates EPAC1 (exchange protein directly activated by cAMP 1), which in turn stimulates ERK (extracellular signal‐regulated kinase) signaling to support thermogenesis. Downstream, PKA promotes thermogenesis by facilitating mitochondrial retention of DRP1 (dynamin‐related protein 1) via TMEM135 (transmembrane protein 135), inhibiting the anti‐thermogenic kinase SIK3 (salt‐inducible kinase 3), and inducing FOXJ3 (forkhead box J3) to enhance thermogenic gene expression. Collectively, these pathways converge on increased thermogenic capacity and expression of genes such as UCP1 (uncoupling protein 1). The figure was created using PowerPoint.

Exposure to cold or the administration of adrenergic receptor agonists is known to activate thermogenesis in brown adipocytes of both rodents and humans [35–38]. During cold exposure, adipose tissue receives sympathetic innervation, leading to the release of norepinephrine. This neurotransmitter binds to *β*‐adrenergic receptors on adipocytes, thereby triggering the Gs signaling pathway and adenylyl cyclase (AC). This activation results in an increase in cAMP levels. Subsequently, cAMP interacts with the regulatory subunit (R) of protein kinase A (PKA), thereby activating and releasing its catalytic subunit (C) to phosphorylate the transcription factor CREB and others. Once phosphorylated, CREB enters the nucleus and upregulates the expression of thermogenic genes, including *Ucp1* [39–42]. The cAMP signaling pathway in adipose tissue metabolism has been extensively reviewed in recent literature [42]. Prolonged cold exposure can induce the browning of sWAT in rodents through an adrenergic stress response. Though PET‐CT analysis did not detect beige adipocytes in human sWAT under cold exposure, burn injury can lead to the formation of UCP1‐positive beige adipocytes within sWAT through an adrenergic stress response. This finding suggests that a white‐to‐beige adipocyte trans‐differentiation is possible in human sWAT [43, 44]. Recent advances in the cAMP pathway, which plays a pivotal role in adipocyte thermogenesis, are briefly encapsulated in Figure 2 and Table 2. The details are as follows.

**Table 2:** Representative regulators of adipocyte thermogenesis and their relevance to obesity. Translational evidence tier definitions: Tier 1 (strong translational potential): Supported by human genetic data (e.g., BMI‐associated variants), human adipose tissue expression data from obese patients, or functional studies in primary human adipocytes. Tier 2 (moderate translational potential): Supported by human adipocyte cell lines, ex vivo human adipose tissue studies, or human fetal BAT data, but lacking in vivo human validation. Tier 3 (preclinical only): Currently supported only by rodent models with no direct human evidence.

Functional category	Regulator	Effect on thermogenesis	In vitro evidence	Animal evidence	Human evidence	Assessment of translational relevance	Ref.
cAMP signaling	ADGRA3	Promote	Yes	Yes	Adipocyte model	Tier 1	[45]
	A2AR	Promote	Yes	Yes	Adipose tissue expression and adipocyte model	Tier 1	[46, 47]
	EDN3–EDNRB	Promote	Yes	Yes	Preadipocyte model	Tier 1	[48, 49]
	EPAC1	Promote	Yes	Yes	Adipocyte model	Tier 1	[50]
	FOXJ3	Promote	Yes	Yes	No direct human evidence	Tier 3	[51]
	AXL	Inhibit	Yes	Yes	Adipose tissue expression and adipocyte model.	Tier 1	[52]
	FPR2	Inhibit	Yes	Yes	Adipocyte model	Tier 1	[53]
	SIK3	Inhibit	Yes	Yes	No direct human evidence	Tier 3	[54]
	AC3‐AT	Inhibit	Yes	Yes	BAT data	Tier 1	[55]
PRDM16–PPAR*γ* axis	CPEB2	Promote	Yes	Yes	Adipose tissue expression	Tier 2	[56]
	IL13	Promote	Yes	Yes	Adipose tissue variation.	Tier 1	[57]
	NR2F6	Promote	Yes	Yes	Fetal BAT and adult omental WAT data	Tier 2	[58]
	PRMT4	Promote	Yes	Yes	Adipose tissue expression	Tier 2	[59]
	TRIM56	Promote	Yes	Yes	Adipose tissue expression	Tier 1	[60]
	YBX3	Promote	Yes	Yes	No direct human evidence	Tier 3	[61]
Mitochondrial function	GCN5L1	Inhibit	Yes	Yes	Human BMI correlation data	Tier 1	[62]
	HIGD1A	Promote	Yes	Yes	No direct human evidence	Tier 3	[63]
	LETMD1	Promote	Yes	Yes	Beige adipocyte model	Tier 2	[64, 65]
	LONP1	Promote	Yes	Yes	Aged human subcutaneous WAT data	Tier 2	[66]
	STEAP4	Promote	Yes	Yes	Adipose tissue expression	Tier 2	[67]
	TMEM135	Promote	Yes	Yes	Adipose tissue expression	Tier 1	[68]
Other regulators	CLCF1	Inhibit	Yes	Yes	Human genetic evidence	Tier 1	[69]
	GPR84	Promote	Yes	Yes	BAT data	Tier 2	[70]
	H1.2	Inhibit	Yes	Yes	Adipose tissue expression	Tier 2	[71]

### 3.1. Positive Regulators (Stimulate cAMP Production or Downstream Signaling)

#### 3.1.1. ADGRA3: A Gs‐PKA‐CREB Axis Activator Promoting Beige Adipogenesis (Preclinical and Human Adipocyte Models)

Adhesion G‐protein‐coupled receptor A3 (ADGRA3) is identified as a constitutively active orphan receptor highly expressed in human and murine adipocytes, particularly in brown and beige adipose tissue. ADGRA3 signaling promotes adipose thermogenesis and improves metabolic homeostasis through the Gs–PKA–CREB axis. Activation of ADGRA3, either via genetic overexpression or by its potential agonist hesperetin, enhances mitochondrial biogenesis, upregulates UCP1 expression, increases oxygen consumption rate, and stimulates glucose uptake in adipocytes. Conversely, knockdown of *Adgra3* in mice exacerbates diet‐induced obesity, reduces core body temperature, impairs lipid metabolism, and leads to insulin resistance [45]. Importantly, these pro‐thermogenic and anti‐obesity effects were consistently observed in both mouse and human adipocyte models, highlighting ADGRA3 as a promising therapeutic target for treating obesity and its related metabolic disorders.

Translational note: ADGRA3 is supported by Tier 1 evidence (human adipocyte models and expression data), and its activation by the dietary flavonoid hesperetin represents a particularly attractive nutritional intervention strategy.

#### 3.1.2. A2AR: Adenosine‐Mediated cAMP Elevation Enhances Mitochondrial Respiration (Preclinical and Human Genetic Data)

The adenosine A2A receptor (A2AR), a G protein‐coupled receptor (GPCR), has emerged as a key regulator of energy metabolism and adipose tissue function in obesity and metabolic disorders. Highly expressed in brown and beige adipocytes, A2AR activation promotes lipolysis, enhances mitochondrial respiration, and upregulates thermogenic gene expression via the cAMP‐PKA signaling pathway [46]. Conversely, adipocyte‐specific *A2ar* knockout (KO) mice (*A2ar*‐FKO) exhibit reduced cold tolerance, decreased oxygen consumption, increased fat mass and body weight, exacerbated insulin resistance, elevated adipose tissue inflammation, and even hepatic steatosis and steatohepatitis [47]. These findings underscore the essential role of A2AR in adipocytes for maintaining metabolic homeostasis and highlight its potential for treating obesity.

Translational note: A2AR is supported by Tier 1 evidence (human adipose tissue expression and genetic data), and adenosine‐based therapeutic strategies are already under preclinical evaluation for metabolic diseases.

#### 3.1.3. EDN3‐EDRB‐EPAC1: cAMP‐EPAC1‐ERK Cascade Driving Thermogenic Differentiation (Preclinical and Human Preadipocyte Models)

Endothelins (EDNs) are peptides that regulate a variety of physiological functions [72]. Mammals possess two types of EDN receptors: the type A receptor (EDNRA) and the type B receptor (EDNRB). EDN1 and EDN2 exhibit equal affinity for both EDNRA and EDNRB, whereas EDN3 demonstrates lower affinity for EDNRA and primarily activates EDNRB [73]. EDNRB, a GPCR, typically signals through Gs or Gq subunits, thereby initiating intracellular calcium and cAMP signaling pathways [74, 75]. EPAC proteins (Exchange Proteins Activated by cAMP, encoded by *Rapgef3*) act as guanine nucleotide exchange factors with an affinity for cAMP comparable to that of PKA [76, 77].

The EDN3‐EDNRB‐EPAC1 axis is recently regarded as a critical regulator of adipose tissue thermogenesis. EDNRB activation in human white preadipocytes initiates a cAMP‐EPAC1‐ERK cascade that drives thermogenic differentiation, while genetic ablation of *Ednrb* in mice disrupts cold‐induced beige adipogenesis, exacerbating obesity and metabolic dysfunctions [48, 49]. Parallel studies reveal that EPAC1 serves as a downstream effector of EDNRB, with pharmacological EPAC1 activation expanding BAT mass and promoting WAT browning in vivo. Conversely, *Epac1* KO models demonstrate impaired BAT development and reduced UCP1 expression. Clinically, a BMI‐associated *RAPGEF3* variant blunts *β*‐adrenergic‐stimulated brown adipocyte proliferation, suggesting that EPAC1 dysfunction may contribute to metabolic disease susceptibility [50]. Together, these findings position the EDN3‐EDNRB‐EPAC1 axis as a potential target for modulating adipose plasticity.

Translational note: This axis is supported by Tier 1 evidence (human preadipocyte models and BMI‐associated RAPGEF3 variants), with EPAC1 representing a pharmacologically targetable node.

#### 3.1.4. FOXJ3: PKA‐Induced Transcription Factor Activating PGC1*α* and UCP1 (Preclinical Only)

FOXJ3 belongs to forkhead transcription factors, which are characterized by a 110‐amino‐acid monomeric DNA‐binding domain [78]. Cold exposure or *β*3‐adrenergic stimulation triggers catecholamine release, activating the cAMP‐PKA cascade to induce *Foxj3* expression in adipocytes. Adipose‐specific *Foxj3* KO mice exhibit BAT whitening, impaired inguinal WAT (iWAT) browning, and obesity. At the molecular level, FOXJ3 directly binds to and transactivates the promoters of key thermogenic genes, including *Pgc1α* (peroxisome proliferator‐activated receptor‐*γ* coactivators 1*α*), and *Ucp1*, establishing it as a downstream effector of the sympathetic nervous system’s regulation of energy expenditure [51]. However, human study of *FOXJ3* in adipocyte thermogenesis is still lacked.

Translational note: FOXJ3 is currently supported only by Tier 3 evidence (rodent models). Human studies are needed to determine whether FOXJ3 represents a viable therapeutic target.

### 3.2. Negative Regulators (Suppress cAMP Levels or Function)

#### 3.2.1. AXL: PDE3/4‐Mediated cAMP Reduction Inhibits Thermogenesis (Preclinical and Human Evidence)

AXL, a receptor tyrosine kinase (RTK) and a member of the TAM family, is activated by its ligand GAS6 with more potency compared with other TAM family members [79]. The downstream intracellular signaling initiated by the AXL receptor is mediated through components of the insulin signaling cascade, including PI3K and AKT. Global deletion of the *Axl* gene results in a transient reduction in weight gain, whereas the genetic ablation of its endogenous ligand GAS6 leads to a decrease in fat mass under high‐fat diet (HFD) conditions [80, 81].

An inducible, adipocyte‐specific KO of *Axl* has been shown to confer protection against diet‐induced obesity in animal models, characterized by increased adipocyte thermogenesis and elevated energy expenditure. Mechanistically, the activity of phosphodiesterases 3 and 4 (PDE3/4), which are established negative regulators of intracellular cAMP levels, is stimulated by an agonistic AXL receptor antibody. Conversely, specific inhibitors of PDE3 and PDE4 result in elevated intracellular cAMP levels and phenocopy the effects on adipocyte thermogenesis observed with pharmacological AXL receptor inhibitors. The observed effects are attributed to the inhibition of the AXL/PDE3/4 signaling axis. By inhibiting this axis, intracellular cAMP levels are increased, which in turn leads to the activation of the PKA pathway. This activation is crucial as it initiates a cascade of downstream signaling events that can influence adipocyte thermogenesis [52].

Translational note: AXL is supported by Tier 1 evidence (human adipose expression and adipocyte models), and small‐molecule AXL inhibitors are already available for other indications, facilitating repurposing for obesity.

#### 3.2.2. FPR2: Ceramide‐Bound GPCR Suppressing cAMP Signaling (Preclinical and Human Adipocyte Models)

Formyl peptide receptor 2 (FPR2), a GPCR, specifically binds long‐chain ceramides, particularly C16:0. This binding activates FPR2 to initiate G_i_ signaling, leading to reduced intracellular cAMP levels and suppressed thermogenic activity in brown and beige adipocytes. Structural analysis via cryo‐electron microscopy revealed that ceramides bind within FPR2’s orthosteric pocket, with key hydrophobic and polar motifs essential for recognition of the ceramide’s fatty acid chain and sphingoid group. Notably, FPR2 exhibited selectivity for saturated ceramides, excluding very‐long chain or unsaturated variants. Functional studies in either *Adiponectin-*Cre or *Ucp1*‐Cre driven adipocyte‐specific *Fpr2*‐KO mice confirmed that FPR2 is required for ceramide‐induced metabolic suppression, as its ablation restored adipocyte thermogenesis and mitigated obesity [53].

Translational note: FPR2 is supported by Tier 1 evidence (human adipocyte models), and ceramide‐FPR2 signaling represents a mechanistic link between circulating lipids and thermogenic suppression.

#### 3.2.3. SIK3: PKA‐Phosphorylated Kinase Inhibiting Thermogenic Gene Expression (Preclinical Only)

Salt‐inducible kinases (SIKs), members of the AMP‐activated protein kinase (AMPK) family, are integral to hormonal signaling and metabolic regulation across various tissues. The three isoforms—SIK1, SIK2, and SIK3—are widely expressed and have been identified as key regulatory factors in these processes [82]. SIK activation is mediated by phosphorylation within the activation domain by the upstream kinase liver kinase B1 (LKB1), while its internal regulatory domain phosphorylation by protein kinase A (PKA) results in SIK inhibition [83].

The study by Shi et al. provides new insights into the role of SIK3 in thermogenesis, revealing that SIK3 functions as a suppressor of the thermogenic gene program in brown adipocytes. Pharmacological SIK inhibition not only enhances basal *Ucp1* expression but also rescues *Ucp1* induction when PKA signaling is blocked, suggesting SIK3 operates downstream of this pathway. Notably, the PKA phosphorylation domain of SIK3 is critical for this regulation, positioning SIK3 as a nexus between *β*‐adrenergic signaling and adipocyte thermogenesis. SIK3 inhibition, either through pharmacological inhibitors or genetic deletion, enhances the expression of thermogenic genes such as *Ucp1* and *Pgc1α*. This effect is mediated through the activation of histone deacetylase 4 (HDAC4), which can reduce the lysine acetylation of PGC1*α*, thereby promoting its coactivator function [54]. In the future, the function of SIK3 in obesity mouse models and human adipose tissue remains to be further investigated to determine whether it could be a potential target for obesity treatment.

Translational note: SIK3 is currently supported only by Tier 3 evidence (rodent models). Future studies should examine SIK3 expression and function in human adipose tissue.

#### 3.2.4. Truncated AC3 (AC3‐AT): ER‐Localized Inhibitor of cAMP Synthesis (Preclinical and Human BAT Data)

ACs, encoded by the *Adcy* gene family, are pivotal in catalyzing the synthesis of the second messenger cAMP. Among these, AC3 is prominently expressed across various somatic tissues, including adipose tissue, kidney, pancreas, and liver [84]. Research on human populations harboring loss‐of‐function mutations in the *ADCY3* gene has established a correlation between these mutations and severe obesity [85, 86]. Mouse models have substantiated these findings, demonstrating that *Adcy3* deficiency perturbs energy homeostasis [87–89].

A specific truncated isoform of AC3, designated AC3‐AT, is selectively induced by cold exposure and beta‐adrenergic stimulation. This truncated AC3‐AT protein is evolutionarily conserved, being present in brown adipocytes from rodents to humans. The AC3‐AT isoform has emerged as an important endogenous modulator of cAMP signaling through its unique subcellular localization and dominant‐negative function. Unlike canonical ACs, AC3‐AT is retained in the endoplasmic reticulum (ER), where it physically interacts with and sequesters functional AC3, limiting its availability at the plasma membrane for cAMP generation. This regulatory mechanism has significant metabolic consequences, as demonstrated by *Adcy3-at* deficient male mice, which exhibit increased energy expenditure and protection against diet‐induced obesity and associated metabolic disorders. The ER‐localized AC3‐AT represents a novel mechanism of control in cAMP signaling, operating through protein–protein interactions rather than enzymatic activity [55].

Translational note: AC3‐AT is supported by Tier 1 evidence (human BAT data). The dominant‐negative mechanism of AC3‐AT offers a unique approach to enhance cAMP signaling by relieving endogenous inhibition.

Taken together, these findings indicate that recently identified cAMP‐associated regulators influence adipocyte thermogenesis not only through classical adrenergic signaling, but also through broader effects on lipolysis, transcriptional activation, and metabolic adaptation. Collectively, these diverse regulators converge on a central signaling hub, the cAMP‐PKA signalosome (Hub 1), which serves as the primary rapid trigger of thermogenic responses.

## 4. New Regulators in PRDM16‐PPAR*γ* Axis (Transcriptional/Translational Regulation, Preclinical and Human Evidence)

The transcription factor PRDM16 (PR domain‐containing 16) is highly enriched in brown adipocytes relative to those in white adipocytes. The molecular mechanisms by which PRDM16 controls brown and beige adipocyte development and function have been extensively reviewed recently by our group [90]. Deletion of *Prdm16* in brown preadipocytes impedes brown adipogenesis and redirects differentiation towards the muscular lineage. In contrast, ectopic expression of *Prdm16* in myoblasts induces their differentiation into brown adipocytes [17, 91]. The adipogenic conversion of myoblasts to brown adipocytes by PRDM16 is contingent upon the presence of rosiglitazone, a specific agonist for peroxisome proliferator‐activated receptor *γ* (PPAR*γ*). While PPAR*γ* alone can convert myogenic cells into adipocytes, it is insufficient for the differentiation into brown adipocytes, which requires PRDM16 expression. PRDM16 interacts with PPAR*γ*, thereby enhancing its transcriptional activity and driving the differentiation of myoblasts into brown adipocytes [17].

**Figure 3:**
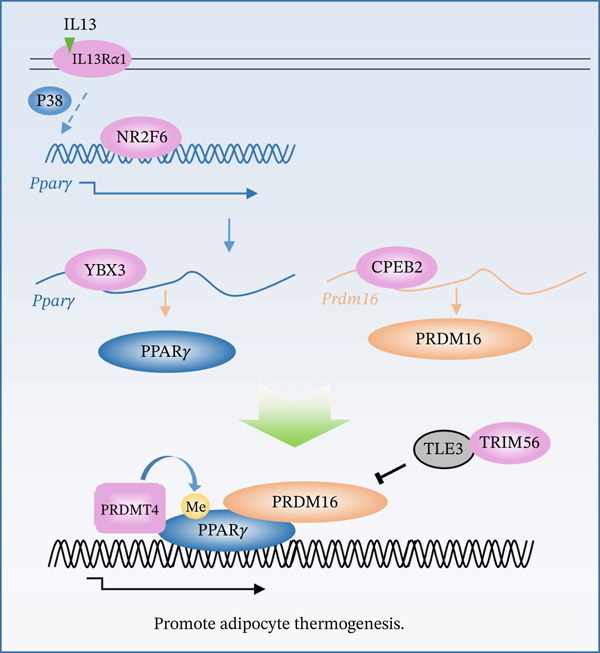
Genes involved in the PR domain containing 16 (PRDM16)‐peroxisome proliferator‐activated receptor gamma (PPAR*γ*) axis regulating adipocyte thermogenesis. This schematic illustrates genes and regulatory factors that enhance adipocyte thermogenesis through the PRDM16‐PPAR*γ* transcriptional axis. Interleukin‐13 (IL‐13) signaling and the orphan nuclear receptor subfamily 2 group F member 6 (NR2F6) converge to induce *Pparγ* transcription. In parallel, Y‐box binding protein 3 (YBX3) promotes thermogenesis by post‐transcriptionally stabilizing *Pparγ* messenger RNA (mRNA). Cytoplasmic polyadenylation element‐binding protein 2 (CPEB2) increases *Prdm16* expression at the translational level, whereas protein arginine methyltransferase 4 (PRMT4) methylates PPAR*γ* and strengthens its interaction with PRDM16. In addition, tripartite motif containing 56 (TRIM56) facilitates thermogenesis by promoting the degradation of transducin‐like enhancer of split 3 (TLE3), thereby preventing its competition with PRDM16 for binding to PPAR*γ*. Collectively, these mechanisms enhance the transcriptional program required for adipocyte thermogenic function. The figure was created using PowerPoint.

*Prdm16* expression levels are elevated in subcutaneous white adipocytes compared with other abdominal white adipocytes in mice. Transgenic expression of *Prdm16* in white fat robustly promotes the development of beige adipocytes in subcutaneous WATs. Mice overexpressing *Prdm16*, particularly on HFDs, exhibit increased energy expenditure, reduced body weight gain, and improved glucose tolerance [92]. In adipose specific *Prdm16* KO mice (Adipo‐*Prdm16* KO), generated by crossing *Prdm16* lox/lox with Adiponectin‐cre mice, beige adipocyte function is significantly inhibited in subcutaneous WAT. Overexpression of *Prdm16* in white adipocytes activates a robust brown fat phenotype and markedly increases uncoupled respiration. PRDM16 directly binds to the promoters of *Ucp1* and *Pgc1α* to drive thermogenic function [91]. The full agonist of PPAR*γ*, rosiglitazone, induces the expression of brown fat genes such as *Ucp1* in primary adipocytes differentiated from the stromal‐vascular fraction (SVF) of inguinal WAT. ShRNA knockdown of *Prdm16* attenuates the effects of PPAR*γ* agonist‐induced browning of inguinal white adipocytes. Conversely, transgenic expression of *Prdm16*, in combination with rosiglitazone, synergistically induces the expression of thermogenic genes and the emergence of UCP1‐positive multilocular adipocytes in vivo. The browning of white adipocytes induced by rosiglitazone is partly regulated through enhanced stability of PRDM16 protein and by inhibiting the ubiquitin‐proteasome pathway [93]. Recently, new findings on the PRDM16‐PPAR*γ* axis have been summarized in Figure 3 and Table 2.

### 4.1. CPEB2: *Prdm16* mRNA Translational Regulator Promoting Brown Adipocyte Function (Preclinical and Human Adipose Expression Data)

Cytoplasmic Polyadenylation Element Binding Protein 2 (CPEB2) is a sequence‐specific RNA‐binding protein that plays a pivotal role in regulating the translation of target mRNAs, thereby influencing a spectrum of physiological processes [94, 95]. Female mice with a complete KO of the *Cpeb2* gene (*Cpeb2* KO) display an increased body mass relative to their control littermates. This phenotype is characterized by a reduction in UCP1 expression and an enlargement of brown adipocytes. Similarly, females with a conditional KO of *Cpeb2* (*Cpeb2* AKO), generated by crossing *Cpeb2* floxed mice with *aP2*‐Cre (*aP2*, also called *Fabp4*, fatty acid binding protein 4) transgenic mice, exhibit significantly elevated body weight and diminished UCP1 expression compared with their controls. Notably, the obese phenotype is more pronounced in female mice than in their male counterparts, although the underlying reasons for this sexual dimorphism remain to be elucidated. The absence of CPEB2 leads to an alteration in the expression of PRDM16 at the protein level, without a corresponding change in its mRNA levels. It is established that CPEB2 binds to *Prdm16* mRNA to regulate its stability and translation. Intriguingly, the ectopic expression of *Prdm16* in the BAT of *Cpeb2*‐KO mice restores thermogenic gene expression profiles and mitigates weight gain [56].

Translational note: CPEB2 is supported by Tier 2 evidence (human adipose expression data). The sexual dimorphism observed in mice (more pronounced in females) warrants investigation in human cohorts.

### 4.2. IL13‐IL13R*α*1 Axis: PPAR*γ* Activation Via STAT6/p38 MAPK (Preclinical and Human IL13RA1 Variant Data)

Interleukin‐13 (IL‐13) and its receptor IL‐13R*α*1 play a crucial role in promoting beige adipogenesis by modulating PPAR*γ* expression via STAT6 and enhancing its activity through p38 MAPK‐mediated PGC1*α* coactivation. In preadipocytes, IL‐13 signaling drives the differentiation of beige adipocytes by augmenting mitochondrial oxidative metabolism and PPAR*γ*‐related pathways. Both IL‐13 and IL‐4 are downstream cytokines of Type 2 innate lymphoid cells (ILC2s) and share a heterodimeric receptor complex composed of IL4R*α* and IL‐13R*α*1 [96]. However, IL‐13’s effects are distinct from those of IL‐4, as IL‐4 KO mice exhibit no defects in beige adipogenesis. This highlights the specificity of the IL‐13/IL‐13R*α*1 axis. Strikingly, *Il13* deficient mice display increased adiposity and body weight. Moreover, genetic analyses have linked human *IL13RA1* variants to body mass index and Type 2 diabetes. These findings advance our understanding of beige adipocyte development and its metabolic significance, positioning the IL‐13/IL‐13R*α*1 signaling pathway as a potential therapeutic target for obesity [57].

Translational note: This axis is supported by Tier 1 evidence (human IL13RA1 genetic variants associated with BMI and T2D), making it a strong candidate for pharmacologic modulation.

### 4.3. NR2F6: Transcriptional Initiator of PPAR*γ* Expression in Brown Adipogenesis (Preclinical and Human Fetal BAT Data)

NR2F6, a member of the nuclear receptor subfamily 2 group F and part of the Chick Ovalbumin Upstream Promoter‐Transcription Factors (COUP‐TFs) family, is an orphan receptor alongside COUP‐TF I (NR2F1), COUP‐TF II (NR2F2), and COUP‐TF III (NR2F6) [97]. NR2F6 promotes fatty acid uptake in the liver, which may contribute to the development of hepatic steatosis and insulin resistance [98]. The nuclear receptor NR2F6 has been recently identified as a differentiation‐stage dependent regulator of brown adipocyte development and function. Exhibiting a dynamic expression pattern, NR2F6 peaks during early brown adipogenesis but declines markedly in mature adipocytes, suggesting distinct roles in progenitor commitment versus terminally differentiated adipocytes. Preadipocyte‐specific *Nr2f6* deficiency disrupts the brown adipogenic program, resulting in impaired thermogenic capacity, and exacerbated metabolic dysfunction under obesogenic conditions. Mechanistically, NR2F6 operates upstream of PPAR*γ* to initiate the brown adipogenesis transcriptional cascade. Notably, this regulatory axis appears specific to BAT, as WAT development remains unaffected by *Nr2f6* deletion. Besides, its expression is highly enriched in human fetal BAT but not omental WAT [58].

Translational note: NR2F6 is supported by Tier 2 evidence (human fetal BAT expression). Its BAT specific role suggests that targeting NR2F6 may have fewer off‐target effects compared to systemic approaches.

### 4.4. PRMT4: PPAR*γ* Methyltransferase Enhancing PRDM16 Interaction (Preclinical and Human Adipose Expression Data)

Protein arginine methyltransferase 4 (PRMT4), also known as coactivator‐associated arginine methyltransferase 1 (CARM1), is a member of the PRMT family. This enzyme plays a critical role in modulating the activity of various transcriptional regulators by methylating arginine residues on its substrates. PRMT4 is implicated in several biological processes, including skeletal muscle differentiation and cell fate determination [99].

PRMT4 exerts a significant influence on adipogenesis and lipid metabolism [100–102]. A recent study has revealed that PRMT4 also serves as a critical regulator of white adipose browning, with its expression upregulated by cold exposure but suppressed in obesity. Overexpression of *Prmt4* in inguinal fat enhances thermogenic gene expression, protects against diet‐induced metabolic dysfunction, and promotes PPAR*γ*‐PRDM16 interaction through methylation of PPAR*γ* at Arg240. Further research should address whether PRMT4‐mediated methylation influences additional thermogenic regulators beyond PPAR*γ* [59].

Translational note: PRMT4 is supported by Tier 2 evidence (human adipose expression data). PRMT4 inhibitors are being developed for cancer; their repurposing for metabolic diseases would require careful evaluation of tissue specificity.

### 4.5. TRIM56: TLE3 Degradation Stabilizing PRDM16‐PPAR*γ* Complexes (Preclinical and Human UCP1 Correlation Data)

Tripartite motif 56 (TRIM56), an E3 ubiquitin ligase, promotes WAT browning and mitigates obesity through the degradation of transducin‐like enhancer protein 3 (TLE3). TLE3 competes with PRDM16 to bind to PPAR*γ*, thereby inhibiting the expression of thermogenic genes. However, TRIM56 can block this competition by promoting the K48‐linked ubiquitination and subsequent degradation of TLE3, thereby enhancing the binding of PRDM16 to PPAR*γ* and promoting the expression of thermogenic genes. Overexpression of *Trim56* in inguinal WAT enhances thermogenic gene expression, increases energy expenditure, and improves metabolic profiles, thereby protecting mice from high fat diet‐induced obesity. The findings are also supported by human data, revealing a positive correlation between *TRIM56* levels and *UCP1* expression, and a negative correlation with body mass index [60].

Translational note: TRIM56 is supported by Tier 1 evidence (human UCP1 correlation and BMI negative correlation). As an E3 ubiquitin ligase, TRIM56 is pharmacologically targetable, though specificity remains a challenge.

### 4.6. YBX3: *Pparγ* mRNA Stabilizer Driving Brown Adipogenesis (Preclinical Only)

The Y‐box binding proteins (YBX1, YBX2 and YBX3) are evolutionarily conserved RNA‐binding proteins that post‐transcriptionally regulate mRNA stability and translation. YBX1 and YBX2 have been shown to facilitate adipocyte thermogenesis by stabilizing the mRNAs of *Pink1* and *Pgc1α*, respectively [103, 104]. More recently, YBX3 was identified as a brown‐adipose‐enriched RNA‐binding protein whose expression is acutely up‐regulated by cold exposure and *β*‐adrenergic signaling. YBX3 plays a critical role in promoting brown adipogenesis and thermogenesis by post‐transcriptionally stabilizing the mRNA of Ppar*γ*. In vivo, BAT‐specific knockdown of *Ybx3* impaired thermogenesis, reduced energy expenditure, and exacerbated diet‐induced obesity and insulin resistance in mice. Conversely, BAT‐specific overexpression of *Ybx3* enhanced thermogenic capacity and protected against metabolic dysfunction under HFD conditions [61].

Translational note: YBX3 is currently supported only by Tier 3 evidence (rodent models). Human adipocyte studies are needed to validate the YBX3‐PPAR*γ* axis.

Collectively, these studies highlight that the PRDM16‐PPAR*γ* axis acts as a central transcriptional platform that integrates diverse upstream cues to control adipocyte identity, thermogenic gene expression, and beige/brown remodeling. We propose that this axis functions as a second major regulatory hub (Hub 2), the transcriptional identity switch, that determines the intrinsic thermogenic capacity of adipocytes.

## 5. New Regulators in Mitochondrial Function (Morphology, Proteostasis, Preclinical and Human Evidence)

Emerging evidence has underscored the significance of mitochondrial morphology and dynamics in regulating the thermogenic function of brown and beige adipocytes [105]. Cold exposure augments BAT glucose uptake and mitochondrial oxidative activity in rodents and humans [106]. Upon cold exposure, mitochondria undergo dynamic remodeling in both morphology and function to meet the energy demands for maintaining body temperature. Mitochondrial morphology is governed by mitochondrial dynamics, which are regulated by specialized proteins belonging to the dynamin‐like family of GTPases, including dynamin‐related protein 1 (DRP1), mitofusins 1 and 2 (MFN1/2), and optic atrophy 1 (OPA1) [107]. DRP1, a cytosolic GTPase, is essential for mitochondrial fission [108]. MFN1/2 are involved in the fusion of the outer mitochondrial membrane [109]. OPA1 is necessary for the fusion of the inner membrane. Conditional KO of *Opa1* in BAT results in fragmented mitochondria and disrupted cristae structure in brown adipocyte [110]. Interestingly, overexpression of *Opa1* converts white adipocytes into beige adipocytes, thereby promoting the browning of WAT and improving systemic glucose tolerance and insulin sensitivity [111]. Mechanistically, OPA1 enhances cAMP‐CREB signaling to upregulate urea cycle enzymes, leading to fumarate accumulation, which synergizes with the chromatin remodeler KDM3A to activate thermogenic genes. This OPA1‐urea cycle axis is essential for beige adipocyte differentiation, as evidenced by impaired browning in *Opa1*‐KO preadipocytes and rescue by fumarate supplementation. Besides, clinical studies reveal obese patients have reduced *OPA1* levels [111]. Moreover, OMA1‐mediated OPA1 processing is crucial for the full activation of BAT thermogenesis, and loss of OMA1 results in obesity and defective thermogenesis [112, 113]. Fission in thermogenic adipocytes is also essential for increased uncoupled respiration by directing fatty acid *β*‐oxidation towards UCP1‐mediated heat production rather than ATP synthesis [113, 114]. Brown adipocyte mitochondria tend to be more fragmented and circular, particularly in response to cold exposure and *β*‐adrenergic receptor stimulation [115]. WAT beiging substantially increases the abundance of numerous mitochondrial proteins involved in cellular respiration, fatty acid oxidation, transmembrane transport, and other essential biological processes to support the needs of efficient transport and consumption of glucose, lipids, amino acids, and other fuel substrates for adipocyte thermogenesis [116, 117]. Therefore, the maintenance of mitochondrial proteome integrity and plasticity is critical to activate and sustain adipocyte thermogenic capacity. Recently, more studies have reported on the role of mitochondrial protein function in adipocyte thermogenesis (Table 2).

### 5.1. GCN5L1: MIC13 Degradation Disrupting Mitochondrial Cristae (Preclinical and Human BMI Correlation Data)

GCN5 Like 1 (GCN5L1), a mitochondrial‐enriched protein known to influence metabolism and dynamics, was found to translocate to the mitochondrial intermembrane space (IMS) in response to a HFD, where it scaffolds protease YME1L and the MICOS component MIC13, promoting MIC13 degradation. MIC13 is a component of the mitochondrial cristae‐organizing system (MICOS) complex and functions in maintaining normal crista structure [118]. This disrupts cristae structure, reduces OXPHOS activity, and impairs energy expenditure in WAT. Conversely, *Gcn5l1* deletion in WAT (but not BAT) enhanced cristae formation, OXPHOS activity, and adipocyte thermogenesis, protecting mice from HFD‐induced obesity. The study also demonstrated that GCN5L1 expression and IMS localization correlate with obesity severity in humans, with higher BMI associated with reduced MIC13 and OXPHOS levels [62].

Translational note: GCN5L1 is supported by Tier 1 evidence (human BMI correlation data). The correlation between higher BMI and reduced OXPHOS levels suggests that GCN5L1 inhibition could be a viable strategy, but tissue specific targeting (WAT vs. BAT) is critical.

### 5.2. HIGD1A: SIRT1 Activation Via NAD^+^/NADH Balance (Preclinical Only)

Hypoxia‐induced gene domain protein‐1a (HIGD1A), a protein localized to the mitochondrial inner membrane, is upregulated in thermogenic adipose tissues in response to cold exposure. Knockdown of *Higd1a* inhibits the browning of adipocytes, while its upregulation fosters this process. Mice with *Higd1a* knockdown in inguinal and brown fat exhibit impaired thermogenesis and are more susceptible to diet‐induced obesity. Conversely, overexpression of *Higd1a* promotes adipose tissue browning, thereby preventing obesity and associated metabolic disorders. Mechanistically, *Higd1a* deficiency impairs mitochondrial respiration, leading to an increase in reactive oxygen species (ROS) levels. Elevated ROS levels enhance the consumption of NAD^+^ for DNA damage repair, reducing the NAD^+^/NADH ratio. This reduction inhibits the activity of sirtuin1 (SIRT1), a key enzyme that facilitates the browning of WAT by PPAR*γ* [119]. Consequently, the inhibition of SIRT1 activity compromises the browning of adipocytes, highlighting the critical role of HIGD1A in maintaining metabolic homeostasis and adipocyte thermogenic function [63].

Translational note: HIGD1A is currently supported only by Tier 3 evidence (rodent models). Human studies are needed to confirm the SIRT1‐NAD^+^ axis in human adipocytes.

### 5.3. LETMD1: Mitochondrial‐Nuclear Shuttle Coordinating Thermogenic Genes (Preclinical and Human Beige Adipocyte Data)

LETMD1 has emerged as a cold‐inducible, BAT enriched protein that critically regulates thermogenesis through multiple interconnected mechanisms. Primarily localized to the mitochondrial matrix, LETMD1 maintains mitochondrial function by preserving cristae structure, supporting OXPHOS complex stability [120, 121], and regulating calcium homeostasis, since its deletion leads to reduced mitochondrial and intracellular Ca^2+^ levels that impair mitochondrial fission and thermogenic capacity [122]. Beyond its mitochondrial role, LETMD1 exhibits stimulus‐dependent nuclear translocation where it interacts with the chromatin remodeler BRG1 (Brahma‐Related Gene 1)/SMARCA4 (SWI/SNF Related, Matrix Associated, Actin Dependent Regulator of Chromatin, Subfamily A, Member 4) to directly activate thermogenic gene programs [123]. This dual compartmentalization allows LETMD1 to synchronize mitochondrial bioenergetics with nuclear transcriptional responses to cold and *β*‐adrenergic stimuli. Genetic studies demonstrate that *Letmd1* KO mice display severe cold intolerance, mitochondrial dysfunction, and impaired adipocyte thermogenesis. Importantly, recent work using proximity labeling techniques has refined our understanding of LETMD1’s molecular topology and interactions in live cells [64]. The therapeutic potential of targeting this pathway is highlighted by findings that pharmacological induction of LETMD1 can enhance energy expenditure and improve metabolic parameters [65]. These collective findings position LETMD1 as a central effector integrating organelle function, calcium signaling, and epigenetic regulation in adaptive thermogenesis.

Translational note: LETMD1 is supported by Tier 2 evidence (human beige adipocyte models). The pharmacologic inducer SP‐8356 has shown promise in preclinical models and represents a lead candidate for translation.

### 5.4. LONP1: Succinate‐Mediated Epigenetic Reprogramming of Beige Adipocytes (Preclinical and Human Aging‐Related Data)

ATP‐dependent proteases are central components of the mitochondrial proteolytic system. Given that approximately two‐thirds of mitochondrial proteins are located within the space of the inner membrane, two ATP‐dependent mitoproteases of the mitochondrial matrix—Lon protease homologue (LONP1) and Clp protease proteolytic subunit (CLPP)—have emerged as key regulators of mitochondrial proteostasis [124, 125].

Mitochondrial protein turnover is significantly elevated during the white‐to‐beige adipocyte conversion in inguinal WAT induced by cold exposure or *β*3 adrenergic receptor agonist treatment. Consistent with the high protein turnover rate associated with white adipocyte browning, LONP1 and CLPP protein levels increase in iWAT, but not in BAT. However, siRNA‐mediated knockdown of *Lonp1*, not *Clpp*, significantly impairs primary beige adipocyte function. Adipocyte‐specific *Lonp1* knockout (*Lonp1* AKO) mice, generated by crossing floxed *Lonp1* mice with *Adipoq*‐Cre transgenic mice, exhibit attenuated multilocular beiging morphological changes and reduced induction of UCP1 in iWAT upon cold exposure or CL316,243 (*β*_3_‐adrenergic receptor agonist) administration. Additionally, *Lonp1* AKO mice demonstrate reduced basal respiration rates and lower body temperature compared to wild‐type (WT) mice.

The mitochondrial protease LONP1 emerges as a critical regulator of white‐to‐beige adipocyte conversion through linking mitochondrial metabolism to epigenetic reprogramming. LONP1 orchestrates this process by selectively degrading succinate dehydrogenase complex iron sulfur subunit B (SDHB), a key component of mitochondrial complex II. This proteolytic regulation maintains elevated intracellular succinate levels, which serve as both a metabolic intermediate and an epigenetic modulator. The accumulated succinate inhibits *α*‐ketoglutarate‐dependent histone demethylases, leading to altered methylation patterns at thermogenic gene loci that promote beige adipocyte differentiation. Importantly, this pathway demonstrates therapeutic potential, as *Lonp1* overexpression can rescue age‐related declines in adipocyte browning capacity [66].

Translational note: LONP1 is supported by Tier 2 evidence (aged human subcutaneous WAT data). The finding that LONP1 overexpression rescues age‐related declines in browning capacity is particularly relevant for an aging obese population.

### 5.5. STEAP4: Mitochondrial Respiratory Complex Regulator (Preclinical and Human Adipose Expression Data)

Six‐transmembrane protein of pros‐tate4 (STEAP4), a metalloreductase, is also known as tumor‐necrosis factor (TNF)‐induced adipose‐related protein (TIARP) or six transmembrane protein of the prostate 2 (STAMP2). *Steap4* is highly expressed in both human and murine adipose tissues. Adipocyte‐specific *Steap4* KO exacerbates HFD‐induced obesity, insulin resistance, and glucose intolerance. Mechanistically, STEAP4 interacts with both mitochondrial proteins and splicing factors, influencing mitochondrial respiratory chain complex activity and alternative splicing of genes involved in mitochondrial function [67].

Translational note: STEAP4 is supported by Tier 2 evidence (human adipose expression data). Its dual role in mitochondrial function and RNA splicing adds complexity to therapeutic targeting.

### 5.6. TMEM135: Peroxisome‐Mitochondria Crosstalk Mediating DRP1 Phosphorylation (Preclinical and Human Obesity Expression Data)

Mitochondrial fission is a complex process that involves the recruitment of multiple organelles, including the ER, and peroxisomes, which are single membrane‐bound organelles integral to lipid metabolism [126, 127]. Peroxisomal biogenesis is upregulated in brown and beige adipocytes in response to cold treatment, a response that is contingent upon the thermogenic protein PRDM16 [128]. Adipose‐specific KO of the critical peroxisomal biogenesis factor *Pex16* (*Pex16*‐AKO), which leads to the absence of peroxisomes in adipose depots, impairs thermogenesis by inhibiting cold‐induced mitochondrial fission due to a reduction in the mitochondrial membrane content of peroxisome‐derived lipids known as plasmalogens [128].

Transmembrane protein 135 (TMEM135) is localized to both peroxisomes and mitochondria and serves as a critical mediator of peroxisomal regulation of mitochondrial fission and thermogenesis [129]. Genetic studies reveal a striking bidirectional phenotype: adipose‐specific *Tmem135* KO mice exhibit impaired mitochondrial fission, reduced thermogenic capacity, and exacerbated diet‐induced obesity, while *Tmem135* overexpression enhances mitochondrial division and protects against obesity and insulin resistance. TMEM135 orchestrates a coordinated response to thermogenic stimuli by mediating the functional crosstalk between peroxisomes and mitochondria. Upon *β*‐adrenergic activation, TMEM135 undergoes plasmalogen‐dependent translocation to mitochondria, where it facilitates PKA‐mediated phosphorylation and mitochondrial retention of DRP1, the master regulator of mitochondrial fission. In humans, *TMEM135* gene expression is decreased in subcutaneous WAT of individuals with obesity [68].

Translational note: TMEM135 is supported by Tier 1 evidence (decreased expression in human obese sWAT). The peroxisome‐mitochondria crosstalk mediated by TMEM135 represents a novel node for intervention.

Overall, these observations emphasize that mitochondrial integrity, dynamics, and proteostasis are indispensable for thermogenic competence, linking organelle quality control directly to adipocyte energy‐dissipating capacity. These mitochondrial factors constitute the third regulatory hub (Hub 3), the metabolic engine, which not only executes thermogenesis but also provides critical feedback to upstream hubs through LETMD1‐mediated nuclear signaling and LONP1 dependent epigenetic reprogramming.

## 6. Other New Regulators (Diverse Mechanisms, Preclinical and Human Evidence)

### 6.1. CLCF1‐STAT3 Signaling: Inhibition of Mitochondrial Biogenesis (Preclinical and Human *CLCF1* Expression Data)

Cardiotrophin‐like cytokine factor 1 (CLCF1) is a member of the interleukin (IL)‐6 family. *CLCF1* expression is downregulated during thermogenic stimulation but is significantly increased in obesity. Adipose tissue‐specific *Clcf1* transgenic (*Clcf1*‐ATG) mice exhibit impaired energy expenditure, reduced mitochondrial biogenesis, severe cold intolerance, and metabolic dysfunction even in the absence of external metabolic stress. Mechanistically, CLCF1 binds to and activates the ciliary neurotrophic factor receptor (CNTFR), thereby enhancing signal transducer and activator of transcription 3 (STAT3) signaling. STAT3 transcriptionally inhibits *Pgc1α* and *Pgc1β*, which suppress mitochondrial biogenesis in brown adipocytes [69]. This is somewhat counterintuitive to the role of IL‐6, which, as demonstrated by Li et al., promotes the beiging of white adipocyte through activating the STAT3 pathway [130]. While both cytokines utilize STAT3 signaling, their effects on thermogenesis appear to be opposite, with CLCF1 acting as a brake and IL‐6 as an activator. This suggests that the interplay between these pathways could be context‐dependent, influenced by the specific adipose tissue type. Future research should focus on elucidating how these pathways interact in different physiological and pathological conditions, as this could provide valuable insights into the regulation of energy homeostasis and potential therapeutic targets for metabolic disorders.

Translational note: CLCF1 is supported by Tier 1 evidence (human genetic data). Its upregulation in obesity positions CLCF1 as an attractive target for therapeutic inhibition. However, the opposing effects of CLCF1 and IL‐6 on thermogenesis, both utilizing STAT3 signaling, underscore the need for pathway selective targeting to avoid interfering with beneficial IL‐6 functions.

### 6.2. GPR84: Medium‐Chain Fatty Acid Receptor Modulating Mitochondrial Ca^2+^ Levels (Preclinical and Human BAT Data)

GPCRs play a crucial role in modulating a broad range of physiological and pathological processes and represent a significant class of drug targets [131]. G protein‐coupled receptor 84 (GPR84) is identified as a receptor for medium‐chain fatty acids [132, 133]. The levels of medium‐chain fatty acids vary under diverse metabolic conditions, such as starvation, cold exposure, and obesity [134–136].

Recent research indicates that GPR84 is highly expressed in BAT and its expression is upregulated by cold stimulation. Mice deficient in *Gpr84* show increased lipid accumulation in BAT, are more susceptible to cold exposure, and exhibit reduced BAT activity compared with their WT counterparts. Primary brown adipocytes from *Gpr84*‐KO mice demonstrate decreased expression of thermogenic genes and reduced oxygen consumption in vitro. Activation of GPR84 induces an increase in intracellular Ca^2+^ levels, which intricately influences mitochondrial respiration. By modulating mitochondrial Ca^2+^ levels and respiration, GPR84 emerges as a potent molecule involved in BAT activity [70].

Translational note: GPR84 is supported by Tier 2 evidence (human BAT data), but functional validation in primary human brown adipocytes is still needed. While medium‐chain fatty acids are natural ligands, GPR84 also has proinflammatory functions in immune cells, so tissue specific targeting would be essential for safe translation.

### 6.3. H1.2‐IL10R*α* Axis: Epigenetic Suppression of Adrenergic Signaling (Preclinical and Human Obese Adipose Data)

Histone variant H1.2 is the most conserved among the five somatic histone H1 variants (H1.1–H1.5) [137]. It plays significant roles in cellular processes such as apoptosis, autophagy, DNA damage, and tumorigenesis [138, 139]. Recent studies have elucidated the role of H1.2 in adipocyte thermogenesis. *H1.2* is enriched in beige and brown adipocytes and is sensitive to temperature changes. Male mice with adipocyte‐specific *H1.2* deletion (*H1.2* AKO) exhibit enhanced energy expenditure, promoted white adipocyte browning, and improved cold tolerance, while overexpression of *H1.2* yields opposite effects. Mechanistically, H1.2 binds to the promoter of the *Il10rα* gene, encoding a subunit of the IL‐10 receptor, and positively regulates its expression to suppress thermogenesis in a beige cell autonomous manner. IL‐10, an anti‐inflammatory cytokine secreted by immune cells including macrophages, B cells, and T cells, inhibits adrenergic signaling and adipose thermogenesis through its receptor Il10r*α* in mature adipocytes [140, 141]. Overexpression of *Il10rα* in inguinal WAT abolishes the cold‐enhanced browning observed in *H1.2* AKO male mice. Increased levels of H1.2 are also found in the WAT of obese humans [71].

Translational note: H1.2 is supported by Tier 2 evidence (increased levels in obese human adipose tissue). As a ubiquitously expressed histone variant, systemic H1.2 inhibition may have broad epigenetic consequences. Adipose targeted delivery approaches would likely be required for safe translation.

Collectively, these emerging pathways further illustrate that adipocyte thermogenesis is governed by a highly interconnected regulatory network, underscoring the need for an integrated perspective across signaling, transcriptional, and mitochondrial mechanisms. This convergence of mechanisms suggests that adipocyte thermogenesis should be interpreted not as a series of isolated pathways, but as a coordinated regulatory system with important unresolved mechanistic and translational implications.

## 7. An Integrated Hub and Network Model of Thermogenic Regulation

**Figure 4:**
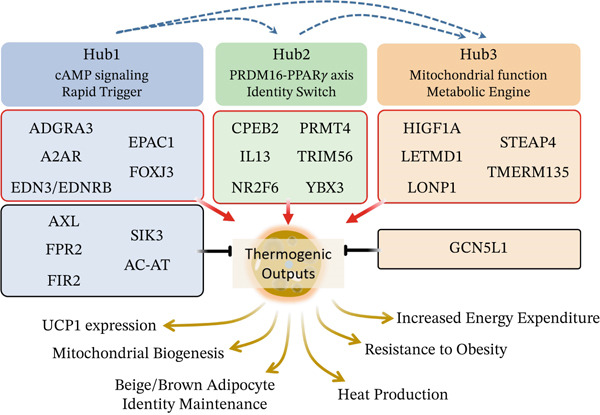
Integrated hub and network model of adipocyte thermogenesis regulation. Three interconnected regulatory hubs control thermogenic capacity in brown and beige adipocytes. Hub 1 (cAMP‐PKA signalosome) serves as the rapid trigger of thermogenesis. Positive regulators, including ADGRA3, A2AR, EDN3/EDNRB, EPAC1, and FOXJ3, activate the Gs‐cAMP‐PKA axis, while negative regulators, including AXL, FPR2, SIK3, and AC3‐AT, suppress cAMP production or downstream signaling. Downstream outputs include lipolysis to provide fatty acids to Hub 3, CREB‐mediated transcription to activate Hub 2, and TMEM135‐DRP1 mediated mitochondrial fission to regulating Hub 3 dynamics. Hub 2 (PRDM16‐PPAR*γ* transcriptional complex) serves as the identity switch that determines thermogenic adipocyte cell fate. It integrates inputs from Hub 1. Positive modulators, including CPEB2, IL13, NR2F6, PRMT4, and TRIM56, enhance complex activity. Outputs include transcription of *Ucp1*, which are delivered to Hub 3. Hub 3 (mitochondrial interface) serves as the metabolic engine that executes UCP1 mediated heat production. It receives fatty acid substrates from Hub 1 and UCP1 from Hub 2. Regulators include HIGD1A, LETMD1, LONP1, STEAP4, TMEM135 (positive) and GCN5L1 (negative). Feedback loops from Hub 3 to Hub 2 operate via LETMD1 and LONP1. The coordinated activity of all three hubs converges on increased UCP1 expression, mitochondrial biogenesis, beige/brown adipocyte identity maintenance, heat production, energy expenditure, and resistance to obesity.

Although the regulators discussed above are presented in distinct categories for clarity, their functions are not independent. We propose that adipocyte thermogenesis is governed by three interconnected regulatory hubs that together determine the magnitude, duration, and sustainability of energy expenditure (Figure 4).

### 7.1. Hub 1: The cAMP‐PKA Signalosome as the Rapid Trigger

This hub serves as the primary sensor of sympathetic and paracrine stimuli. It integrates signals from multiple GPCRs and RTKs to generate dynamic cAMP pulses that are decoded by PKA and EPAC. Molecules such as ADGRA3, A2AR, and EDNRB converge to activate this hub, while AXL, FPR2, and AC3‐AT act as endogenous brakes. Downstream effectors including PKA, EPAC1, and SIK3 translate cAMP dynamics into rapid changes in lipolysis, mitochondrial dynamics via the TMEM135‐DRP1 axis, and immediate early transcription via CREB and FOXJ3. The defining feature of this hub is its speed and reversibility, making it ideal for acute responses to cold or feeding status. However, its chronic activation in obesity may lead to desensitization and downregulation, limiting its therapeutic window.

### 7.2. Hub 2: The PRDM16‐PPAR*γ* Transcriptional Complex as the Identity Switch

This hub determines the cell’s intrinsic thermogenic capacity and represents the master regulator of brown/beige adipocyte identity. It operates on a slower timescale, controlling the expression of *Ucp1*, *Pgc1α*, and hundreds of other thermogenesis related genes. The hub integrates inputs from Hub 1 via CREB‐mediated transcription, from immune‐metabolic axis (e.g., IL13‐STAT6), and from lineage‐determining factors (e.g., NR2F6, YBX3). Regulators such as CPEB2, which stabilizes *Prdm16* mRNA, PRMT4, which methylates PPAR*γ* to enhance PRDM16 interaction, and TRIM56, which degrades the inhibitor TLE3, all enhance the stability and activity of the PRDM16‐PPAR*γ* complex. Conversely, TLE3 competes with PRDM16 for PPAR*γ* binding, suppressing thermogenic gene expression. The state of this hub defines whether an adipocyte adopts a white, beige, or brown phenotype. Importantly, obesity associated inflammation and metabolic stress can epigenetically silence this hub, leading to the whitening of brown and beige adipose tissue.

### 7.3. Hub 3: The Mitochondrial Interface as the Metabolic Engine

This is the final effector of thermogenesis, where UCP1 mediated uncoupling of oxidative phosphorylation generates heat. The performance of this hub depends on two major inputs: (1) from Hub 1, which supplies fatty acid substrates via lipolysis and triggers mitochondrial fission (via TMEM135‐mediated DRP1 phosphorylation); and (2) from Hub 2, which supplies the protein machinery, including UCP1, PGC1*α*, and components of the electron transport chain. (3) Beyond its role as a passive effector, Hub 3 actively communicates back to the other hubs. LETMD1 exhibits stimulus‐dependent nuclear translocation where it interacts with the chromatin remodeler BRG1 to directly activate thermogenic genes. LONP1 degrades SDHB to elevate succinate levels, which inhibits histone demethylases and epigenetically reinforces the beige adipocyte program. GCN5L1 disrupts cristae structure and impairs thermogenesis when dysregulated, while STEAP4 and HIGD1A maintain mitochondrial respiratory capacity and redox balance. Collectively, these feedback mechanisms establish a mitochondria‐to‐nucleus signaling axis that can either reinforce or suppress the thermogenic program over longer timescales.

### 7.4. Network Properties and Emerging Knowledge Gaps

This hub based framework explains several puzzling observations in the literature. First, convergence, the fact that many different KO mice exhibit cold sensitivity or obesity, occurs because diverse regulators ultimately impinge on the same core hubs. Second, redundancy, the existence of multiple positive and negative regulators within each hub, provides robustness but also complicates therapeutic targeting, as inhibiting a single negative regulator may be compensated by others. Third, feedback, particularly from Hub 3 to Hubs 1 and 2, suggests that thermogenic failure in obesity is likely a self‐reinforcing state: mitochondrial dysfunction (Hub 3) may actively suppress the transcriptional identity (Hub 2) and signaling responsiveness (Hub 1), creating a vicious cycle.

At the same time, several important questions remain unresolved. First, many mechanistic studies have been conducted in murine adipocyte models, whereas human thermogenic adipose depots differ in anatomical distribution and inducibility, which may limit direct translation of rodent findings. Second, although numerous molecules enhance thermogenic markers in cultured adipocytes or improve metabolic phenotypes in obese mice, their long‐term efficacy, safety, and tissue specificity in humans remain uncertain. Third, the induction of thermogenic genes alone may not be sufficient to achieve durable metabolic benefit, because adipocyte thermogenic activity is also strongly influenced by mitochondrial quality, vascularization, inflammatory status, and adipose tissue plasticity [142, 143]. In addition, emerging evidence suggests that prior obesity may leave persistent molecular or epigenetic alterations in adipose tissue, raising the possibility that some thermogenic defects are only partially reversible [144, 145]. These issues highlight the need for future studies that integrate molecular mechanisms with physiological context and translational relevance.

Importantly, we emphasize that not all mechanisms discussed are equally ready for translation. While Tier 1 targets (ADGRA3, A2AR, EDNRB‐EPAC1, AC3‐AT, TRIM56, LONP1, CLCF1, TMEM135) are supported by human evidence and represent the most immediate opportunities, Tier 2 and Tier 3 mechanisms require further validation in human systems. Future translational efforts should prioritize these Tier 1 targets while continuing to validate promising preclinical mechanisms in human adipocyte models and tissue cohorts.

## 8. Translational Relevance and Current Limitations

The growing understanding of adipocyte thermogenesis has created multiple opportunities for obesity treatment, but several translational challenges must be considered. Most mechanistic insights have been obtained from rodent models, particularly murine interscapular BAT and inducible beige adipocytes in sWAT. Although these models have provided fundamental knowledge, human thermogenic adipose tissue differs from rodent fat in anatomical distribution, cellular composition, thermogenic capacity, and responsiveness to external stimuli [146, 147]. In adult humans, thermogenic adipocytes are predominantly detected in supraclavicular, cervical, paravertebral, and perirenal regions, and many depots appear to contain mixed populations with both brown‐ and beige‐like characteristics [148, 149]. These interspecies differences suggest that molecular targets identified in rodents may not always exert equivalent effects in humans.

### 8.1. Prioritizing Mechanisms With the Strongest Translational Potential

To bridge the preclinical to clinical gap, we have systematically classified all regulators discussed in this review based on the strength of human evidence. Based on this classification, we identify the following mechanisms as having the strongest translational potential (Tier 1):

ADGRA3 (Hub 1). Supported by human adipocyte models and expression data. The identification of hesperetin as a potential agonist offers a nutritional intervention strategy that could be rapidly tested in human trials.

A2AR (Hub 1). Supported by human adipose tissue expression and genetic data. Adenosine‐based therapies are already being evaluated for other indications, facilitating repurposing for obesity.

EDNRB‐EPAC1 axis (Hub 1). Supported by human preadipocyte models and BMI‐associated RAPGEF3 variants. EPAC1‐selective activators are under development and could enhance BAT mass and WAT browning.

AC3‐AT (Hub 1). Supported by human BAT data. The dominant‐negative mechanism offers a unique approach to enhance cAMP signaling by relieving endogenous inhibition, rather than directly activating receptors.

TRIM56 (Hub 2). Supported by human UCP1 correlation and BMI negative correlation. As an E3 ubiquitin ligase targeting TLE3, TRIM56 is pharmacologically targetable, though isoform selectivity remains a challenge.

LONP1 (Hub 3). Supported by aged human subcutaneous WAT data. The demonstration that LONP1 overexpression rescues age‐related declines in browning capacity is particularly relevant for the aging obese population.

TMEM135 (Hub 3). Supported by decreased expression in human obese sWAT. The peroxisome‐mitochondria crosstalk mediated by TMEM135 represents a previously unrecognized node for intervention.

CLCF1 (Hub 3). Supported by human genetic evidence. As a negative regulator, CLCF1 inhibition represents a strategy to disinhibit thermogenesis, though potential off‐target effects on other STAT3‐dependent processes require careful evaluation.

In contrast, mechanisms currently supported only by Tier 3 (preclinical) evidence, including FOXJ3, SIK3, YBX3, HIGD1A, and GCN5L1, require validation in human adipocyte models or human tissue cohorts before they can be considered for therapeutic development.

### 8.2. Remaining Translational Barriers

In addition to the evidence gap, the therapeutic activation of adipocyte thermogenesis remains complex in the setting of obesity. Many candidate regulators enhance UCP1 expression, mitochondrial activity, or oxygen consumption in cultured adipocytes or improve metabolic phenotypes in diet‐induced obese mice. However, acute induction of thermogenic markers does not necessarily predict sustained clinical benefit [150]. The long‐term efficacy of thermogenic interventions is likely influenced by multiple obesity‐associated abnormalities, including inflammation, mitochondrial dysfunction, impaired vascularization, reduced adipose plasticity, and whitening of brown and beige fat [29]. Therefore, successful translational strategies may need not only to activate thermogenesis, but also to restore the structural and functional integrity of thermogenic adipose tissue.

Safety and specificity also remain important concerns. Many pathways that stimulate adipocyte thermogenesis, particularly those linked to sympathetic activation, may have off‐target effects on the cardiovascular system or other organs [151, 152]. For this reason, future therapies will require improved tissue selectivity and more precise control of thermogenic activation. In parallel, recent evidence suggesting that adipose tissue may retain a molecular or epigenetic memory of prior obesity raises an additional challenge: some defects in adipose thermogenic function may persist even after weight reduction. Collectively, these issues indicate that the most promising therapeutic approaches will likely be those that combine mechanistic specificity, metabolic durability, and human relevance, with the Tier 1 targets identified above representing the most immediate opportunities for translation.

## 9. Conclusion and Future Perspectives

Recent advances in cAMP‐centered signaling, PRDM16‐PPAR*γ*‐directed transcriptional control, and mitochondrial quality‐control mechanisms have revealed several convergent regulatory nodes that may be therapeutically actionable. Together, these findings indicate that thermogenic regulation is governed not by isolated molecules, but by interconnected networks that collectively shape adipocyte identity, mitochondrial competence, and metabolic function.

Despite this progress, important challenges remain for clinical translation. Many mechanistic insights have been derived from rodent models, whereas human thermogenic adipose tissues differ in anatomical distribution, cellular composition, inducibility, and metabolic responsiveness [150, 153]. In addition, obesity‐associated adipose dysfunction, including inflammation, mitochondrial impairment, reduced plasticity, and possible persistent molecular memory, may limit the durability of thermogenic interventions [154, 155]. Therefore, the induction of thermogenic genes alone may not be sufficient to achieve sustained metabolic benefit.

Future progress in this field will depend on integrating mechanistic, physiological, and translational studies to identify regulatory nodes that are effective, tissue‐selective, and relevant in humans. A deeper understanding of shared pathways, context‐dependent regulation, and irreversible or partially reversible obesity‐induced alterations will be essential for advancing adipocyte thermogenesis from an experimental concept toward a realistic therapeutic strategy for obesity treatment.

**Abbreviations**
A2AR: adenosine A2A receptor
AC: adenylyl cyclase
ADGRA3: adhesion G‐protein‐coupled receptor A3
BAT: brown adipose tissue
cAMP: cyclic adenosine monophosphate
CLCF1: cardiotrophin‐like cytokine factor 1
CPEB: cytoplasmic polyadenylation element binding protein
EDN: endothelins
EDNR: endothelin receptors
EPAC: exchange proteins activated by cAMP
FPR2: formyl peptide receptor 2
GCN5L1: GCN5 Like 1
GPCR: G protein‐coupled receptors
HFD: high‐fat diet
HIGD1: hypoxia‐induced gene domain protein‐1
IL13: interleukin‐13
LONP1: lon protease homologue 1
NRF2: nuclear factor erythroid 2‐related factor 2
OXPHOS: oxidative phosphorylation
OPA1: optic atrophy 1
PDE: phosphodiesterases
PKA: protein kinase A
PRDM16: PR domain‐containing 16
PPAR*γ*
: peroxisome proliferator‐activated receptor *γ*
PRMT4: protein arginine methyltransferase 4
STEAP4: six‐transmembrane protein of prostate 4
SIK: salt‐inducible kinases
TMEM135: transmembrane protein 135
TET2: ten‐eleven translocation 2
TRIM56: tripartite motif 56
UCP1: uncoupling protein 1
WAT: white adipose tissue
YBX3: Y‐box binding protein 3

## Funding

This study was supported, in part, by the Natural Science Foundation of Guangdong Province (2026A1515011102), the Guangzhou Science and Technology Plan Project (2025A03J3666), Key Project of Department of Education of Guangdong Provincial (2024ZDX2081, 2021JDA029), National Natural Science Foundation of China (81970729), Natural Science Joint Foundation of Hubei Province (2023AFD038), Innovation and Entrepreneurship Training Program for College Students (X202510519103), and Initial Funding of Hubei University of Arts and Science (2059200).

## Conflicts of Interest

The authors declare no conflicts of interest.

## Data Availability

There are no raw data associated with this review article.
